# Perceived importance of pandemic interventions for attending cultural events – findings from Germany

**DOI:** 10.1186/s12889-022-13358-8

**Published:** 2022-05-10

**Authors:** Michaela Weber, Manuel Plew, Christine Neumann, Marietta Ostendorf, Raphael Herr, Joachim Fischer

**Affiliations:** 1 Mannheim Institute of Public Health, Social and Preventive Medicine, Heidelberg University, Ludolf-Krehl-Str. 7-11, 68167 Mannheim, Germany; 2 Reservix GmbH, Frankfurt, Germany

**Keywords:** COVID-19, Coronavirus, Pandemic, Safety, Containment, Events, Relevance, Reopening

## Abstract

**Background:**

During the first waves of the COVID-19 pandemic, many cultural and sporting events were held without spectators or had to be cancelled. Therefore, several containment strategies to provide requirements for safe events were developed and tested. Nonetheless, every second (50.7%) is afraid of becoming infected on an event. We therefore investigated which hygiene and containment measures are perceived to be important from the visitor’s point of view and thus might increase subjective sense of safety.

**Methods:**

This online study was carried out in November 2020. A total of 1,004 persons, who regularly attended events before the pandemic, took part in the study. The importance of different hygiene and containment measures was evaluated using a 5-point Likert-scale (1 “unimportant” to 5 “extremely important”). Potential statistical differences in socio-demographical aspects (age, gender, net disposable income for leisure activities) and attendance on events were tested with analyses of variance.

**Results:**

Participants perceived the use of disinfectant (M = 4.10) as the most important element of containment strategies, followed by transparent information on the hygiene strategy (M = 4.00), reduced occupancy (M = 3.98), and optimized ventilation (M = 3.97). Body temperature measurement at the entrance (M = 3.27), a negative SARS-CoV-2 test (M = 3.11), completion of a health questionnaire (M = 3.05), and abandoning breaks and catering (M = 2.98) were considered as less important.

Analyses of group differences in socio-demographical aspects found abandoning breaks and catering to be more important to men than to women. This strategy is also more important to people aged 66 and above than to younger age groups (e.g., age 20–40). For women, the use of disinfectant is considerably more important. No other significant differences exist.

**Conclusion:**

Combining relevant measures appears to be important to provide a safe containment strategy. Measures aimed at positively influencing people’s sense of safety do not fully correspond to researched knowledge of effectiveness.

There are also target group-specific differences in the rating of measures, which should be considered while preparing containment strategies. To describe the dynamic development of changes in subjective rating of containment strategies, continuing research is needed.

## Background

Opera, theatre, football stadium—the cultural sector has been strongly affected in Germany since the beginning of the pandemic in March 2020. First attempts exist to support an opening of cultural events to the public under appropriate hygiene and containment strategies [1].

Various pilot projects in the event sector have proven that cultural and sporting events with appropriate containment strategies are possible. In addition to pilot projects abroad, noteworthy pilot projects have also taken place in Germany, creating a perspective of reopening for cultural and sports event organizers, e.g., the home match of Germany’s Bundesliga football club Union Berlin on 13 March 2021, with 165 accredited visitors tested negative for SARS-CoV-2. The Berlin Philharmonic Orchestra also conducted a pilot project ("Perspektive Kultur") on 20 March 2021 with 1,000 visitors tested negative [1]. Additionally, on both events, face masks, social distancing, one-way systems as well as reduced occupancy have been part of the safety concept. Previous studies in this area focused on aerosol dispersion at indoor events and transmission pathways and analysed the medical effectiveness of single interventions such as ventilation, social and physical distancing, and the use of face masks [2, 3].

Making both, indoor and outdoor events, accessible to visitors once again during a pandemic, specific hygiene and containment strategies are needed to prevent the spread of the virus, particularly in the context of indoor events. Besides scientific and medical strategies for safety, it is also necessary to consider the visitors’ point of view to identify important measures. This will allow for the derivation of practical implications to optimize existing hygiene and containment strategies and thus increase their acceptance and individual’s subjective feeling of safety. This study therefore aims to answer the question which hygiene and containment strategies are seen to be most important to provide an indication for organizing and attending future cultural events in a safe manner during a pandemic. By comparing the different aspects in terms of age, gender and net disposable income, this study fills a significant gap in the current state of knowledge, as there are, to the best of our knowledge, no other studies that compare different components of hygiene strategies and, above all, investigate which measures are supported by the visitors and which increase or decrease an individual`s feeling of safety.

## Methods

An online survey with 1,004 participants, who regularly attended cultural, sports and music events before the pandemic, was conducted from November 16 to 20, 2020, in Germany. The target group was randomly selected throughout Germany, i.e., addressed by E-Mail through a market research institute (Respondi AG) on behalf of the German ticketing company Reservix. The sample was stratified by equal gender and age groups to enable the detection of potential subgroup differences. Respondents answered questions on reasons why not attending events as well as on the perceived relevance of hygiene and containment measures. Each item was assessed by a 5-point Likert Scale, ranging from 1 “unimportant” to 5 “extremely important”. Other responses (e.g., “not applicable” or “don’t know”) were not possible. All ten items measure the latent construct of covid measurement well (Cronbach’s alpha = 0.90).

The aspects included the following protective measures already used in many places and recommended by the Robert Koch Institute (RKI), the German government`s central scientific institution in the field of biomedicine [4] as well as the World Health Organization (WHO) [5]:

- Washing hands
- Transparent information about the hygiene strategy used
- Reducing occupancy
- Ventilation concept (e.g., professional ventilation system; adequate supply of outdoor air in indoor event locations)
- Use of face masks
- Personalized tickets (registering attendees)
- Body temperature measurements at the entrance
- Negative SARS-CoV-2 test (not older than 24 h)
- Health questionnaire
- Abandoning breaks and catering.

In addition, we collected socio-demographic information to differentiate the assessed attributes with respect to respondents, i.e., gender, age, net disposable income for leisure activities per month, and (not) attending any event (i.e., indoor/outdoor; large/small audience; different seasons) during the pandemic (since April to November 2020). Possible group differences for perceived relevance in hygiene and containment measures were tested using one-way analyses of variance. If necessary, variables were transformed to approach normal distribution.

## Results

A total of 1,004 persons (50% female), who regularly (e.g. once a month) attended cultural or sporting events before the pandemic, participated in the study. Most respondents (31.5%) reported having more than 500 Euros per month to spend on leisure activities, and pre-pandemic, pop/rock concerts were most frequently attended (49.4%) (Table 1). All the participants stated that they had bought tickets for cultural, sports or music events at least once in the last 24 months.

**Table 1:** Money for leisure activities and event attendance

	%	n
Money available		
< = 50 €	10,7%	107
51—100 €	13,1%	132
101—200 €	19,8%	199
201—500 €	24,9%	250
> 500 €	31,5%	316
Event attendance before pandemic		
Pop / rock concerts	49,4%	496
Classical music	18,5%	186
Musical	25,9%	260
Theatre	35,4%	355
Cabaret / comedy	27,1%	272
Shows	21,3%	214
Football	26,8%	269
Other sport events	13,4%	135
None of the above	5,2%	52

The most often cited reason for not attending or rarely attending events was for fear of infecting themselves (50.7%), with 70.6% of them saying that they would, at least sometimes, avoid events due to the fear of infection, while 11.1% of them claiming to feel safe.

**Fig. 1:**
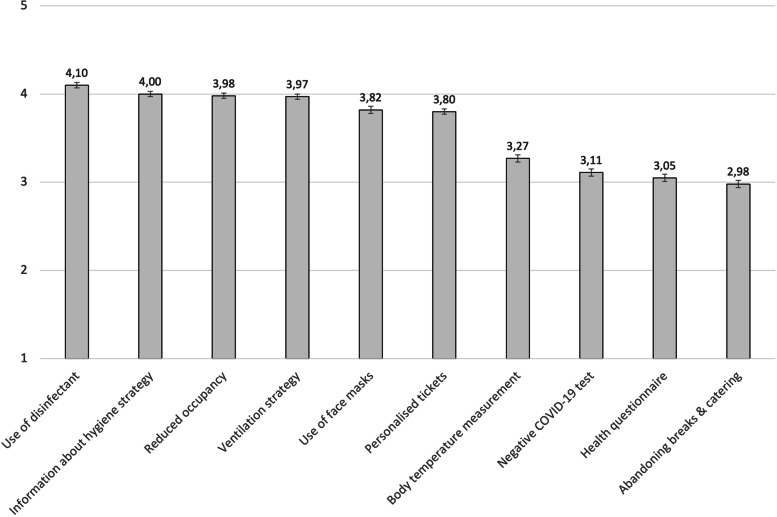
Importance of aspects to attend cultural events**.** Legend: 1 = unimportant to 5 = extremely important. Mean values and standard error

In terms of importance regarding hygiene measures and containment concepts, the use of disinfectants was of utmost importance, followed by transparent information about the hygiene strategy. In contrast, a negative SARS-CoV-2 test, completing a health questionnaire or abandoning breaks and/or catering was of least importance (see Fig. 1).

To identify group differences, aspects were examined in terms of gender, age (categories), net disposable income for leisure activities per month, and attendance of events during the pandemic (since April to November 2020).

**Fig. 2:**
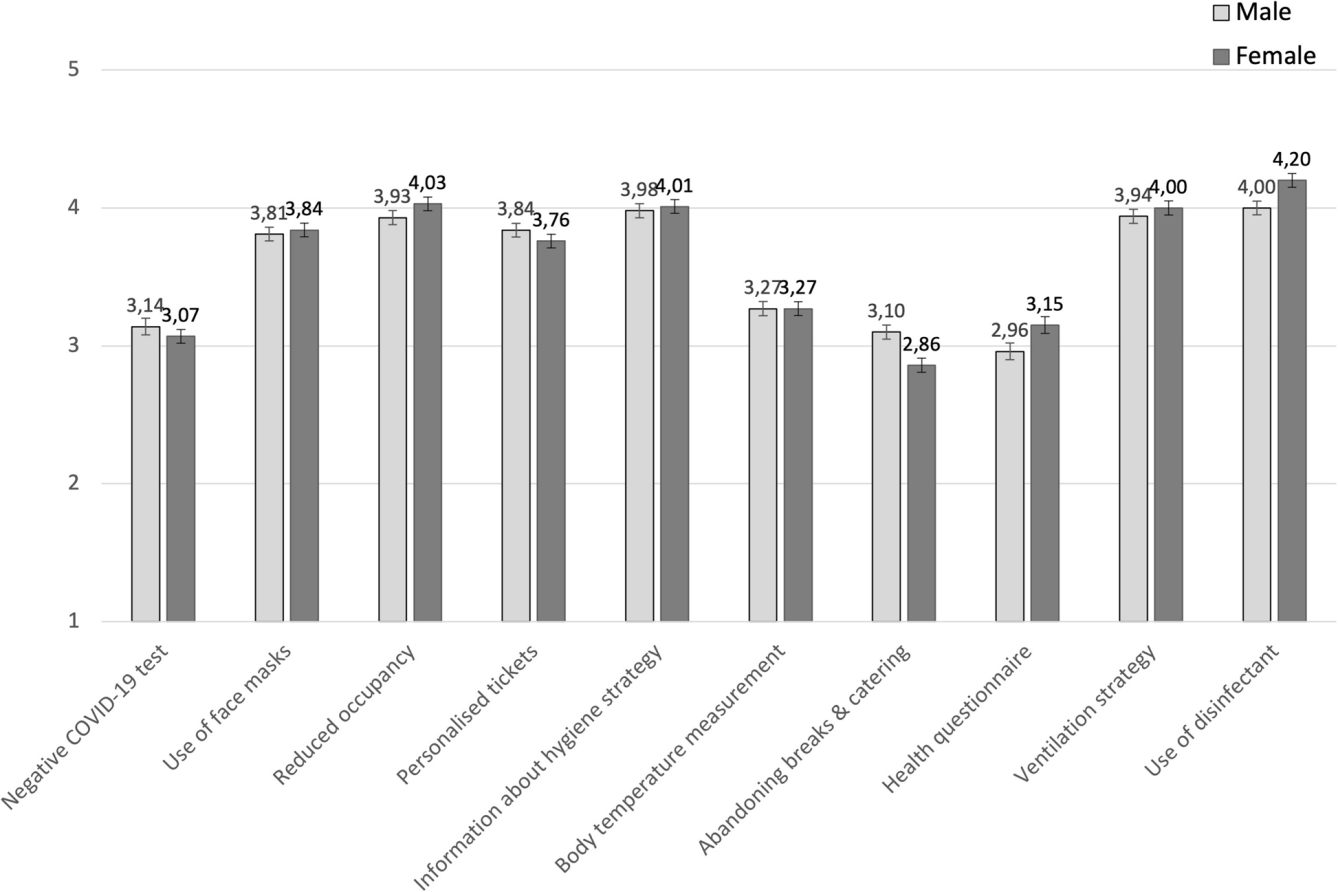
Gender differences in the importance of attending cultural events**.** Legend: 1 = unimportant to 5 = extremely important. Mean values and standard error. ** *p* ≤ 0.001; * *p* ≤ 0.05

Regarding the gender distribution, the subjective relevance of hygiene and containment measures differs only in a few aspects (Fig. 2), with men considering an abandonment of breaks and catering significantly more important than women (M_men_ = 3.10, M_women_ = 2.86; F(1,999) = 10.152, *p* = 0.001). Although the use of disinfectants is of utmost importance to men and women, it is significantly more important to women (M_men_ = 4.00, M_women_ = 4.20; F(1,999) = 11.944, *p* = 0.001).

Regarding the age of respondents, several significant differences could be found (see Table 2). Particularly for abandoning breaks and catering, the use of disinfectants, and the importance of filling out a health questionnaire differences in relevance depending on age were observed (all *p*- values < 0.001).

**Table 2:** Importance of aspects according to age groups

	< 18 (*n* = 145)		18—21 (*n* = 145)		21—35 (*n* = 147)		36—50 (*n* = 145)		51—65 (*n* = 145)		66–75 (*n* = 130)		> 75 (*n* = 130)		*F*-value	*P*-value
	Mean	SE	Mean	SE	Mean	SE	Mean	SE	Mean	SE	Mean	SE	Mean	SE		
Negative SARS-CoV-2 test	3,23	0,10	3,33	0,10	2,83	0,10	2,90	0,11	3,06	0,11	3,07	0,11	3,35	0,09	3,88	0,001
Use of face masks	3,86	0,09	3,74	0,10	3,65	0,11	3,63	0,11	3,89	0,09	3,99	0,10	4,04	0,09	2,34	0,030
Reduced occupancy	3,97	0,08	4,01	0,09	3,90	0,10	3,89	0,10	4,10	0,09	4,04	0,09	3,97	0,09	0,63	0,703
Personalised tickets	3,73	0,09	3,62	0,08	3,75	0,10	3,62	0,10	4,01	0,09	3,93	0,09	3,95	0,09	3,15	0,005
Information on the hygiene strategy	4,13	0,08	4,07	0,08	3,90	0,10	3,89	0,10	3,98	0,09	3,95	0,09	4,06	0,08	0,88	0,512
Body temperature measurement	3,25	0,09	3,33	0,09	3,16	0,10	3,19	0,11	3,35	0,10	3,29	0,10	3,35	0,10	0,64	0,696
Abandoning breaks & catering	2,61	0,09	2,69	0,09	2,67	0,09	2,96	0,10	3,03	0,10	3,43	0,09	3,50	0,10	14,46	< 0,001
Health questionnaire	3,48	0,09	3,23	0,10	2,93	0,11	2,88	0,11	2,92	0,11	3,03	0,10	2,90	0,10	4,82	< 0,001
Ventilation strategy	3,88	0,08	4,07	0,08	4,01	0,09	3,87	0,10	3,99	0,09	3,95	0,09	4,05	0,08	0,74	0,614
Use of disinfectant	4,27	0,08	4,30	0,07	4,14	0,10	3,81	0,10	4,03	0,09	3,98	0,09	4,17	0,08	3,87	0,001

The net income available per month for leisure activities differs significantly with respect to perceived importance of negative SARS-CoV-2 test, use of face masks, personalised tickets, abandonment of breaks and catering, health questionnaire, and use of disinfectant (all *p*-values < 0.001) (Table 3). Especially those with little money for leisure activities per month (≤ 50 euros) considered the abandonment of breaks and catering to be less important than the other respondents with higher net income per month available for leisure activities.

**Table 3:** Importance of aspects according to money available

	< 50 € (*n* = 107)		51—100 € (*n* = 132)		101—200 € (*n* = 199)		< 201—500 € (*n* = 250)		> 500 € (*n* = 316)		*F*-value	*P*-value
	Mean	SE	Mean	SE	Mean	SE	Mean	SE	Mean	SE		
Negative SARS-CoV-2 test	3,06	0,12	3,27	0,11	3,17	0,09	3,13	0,08	3,00	0,07	1,40	0,232
Use of face masks	3,67	0,12	4,03	0,09	3,89	0,08	3,82	0,07	3,76	0,07	1,58	0,178
Reduced occupancy	3,86	0,11	4,16	0,08	4,00	0,08	3,99	0,06	3,93	0,07	1,18	0,318
Personalised tickets	3,50	0,11	3,88	0,08	3,85	0,08	3,83	0,07	3,81	0,06	2,28	0,059
Information on the hygiene strategy	3,81	0,10	4,21	0,08	4,13	0,07	3,94	0,06	3,93	0,06	3,93	0,004
Body temperature measurement	3,08	0,10	3,40	0,10	3,33	0,08	3,38	0,08	3,16	0,07	2,48	0,042
Abandoning breaks & catering	2,50	0,10	2,91	0,09	2,97	0,08	3,12	0,07	3,05	0,07	6,02	< 0,001
Health questionnaire	3,07	0,12	3,39	0,10	3,11	0,09	2,99	0,08	2,92	0,07	3,68	0,006
Ventilation strategy	3,89	0,10	4,05	0,08	3,99	0,07	3,98	0,06	3,95	0,06	0,35	0,847
Use of disinfectant	4,06	0,11	4,30	0,08	4,07	0,07	4,14	0,07	4,01	0,06	1,87	0,114

With respect to differences between respondents who attended and those who did not attend an event during the COVID-19 pandemic (Table 4), only the importance of a negative SARS-CoV-2 test was seen as being more important to those who did not attend events during the pandemic as compared to those who attended events (F(1,1002) = 13.374, *p* < 0.001).

**Table 4:** Importance of aspects according to attendance

	No visit (*n* = 720)		Visit (*n* = 284)		*F*-value	*P*-value
	Mean	SE	Mean	SE		
Negative SARS-CoV-2 test	3,20	0,05	2,88	0,07	13,37	< 0,001
Use of face masks	3,82	0,05	3,84	0,07	0,01	0,924
Reduced occupancy	3,95	0,04	4,07	0,06	1,76	0,185
Personalised tickets	3,80	0,04	3,81	0,07	0,03	0,855
Information on the hygiene strategy	3,99	0,04	4,02	0,06	0,10	0,748
Body temperature measurement	3,29	0,04	3,22	0,07	0,80	0,371
Abandoning breaks & catering	3,01	0,04	2,89	0,07	1,96	0,162
Health questionnaire	3,06	0,05	3,05	0,07	0,01	0,930
Ventilation strategy	3,96	0,04	4,00	0,06	0,38	0,538
Use of disinfectant	4,07	0,04	4,15	0,06	1,00	0,317

## Discussion

Study results suggest that the importance of hygiene and containment measures are rated differently, also depending on socio-demographical aspects. Especially the importance of a transparent hygiene strategy was highlighted by respondents, showing that declarations on the hygiene strategy and appropriate, comprehensible communication of it provide trust and a feeling of safety.

Unexpectedly, a negative SARS-CoV-2 test was, among other things, rated least important although various authors emphasize the necessity of targeted and systematic testing of asymptomatic persons to break the chain of infection [6, 7]. The low relevance might be explained by respondents’ mistrust in self-assessments and self-tests due to threat of false statements and/or manipulated test results, thus being not reliable. Findings of previous studies also show that overly high costs and effort reduce individual`s willingness for testing [8].

Thus, the ubiquitous wearing of effective face masks is very important to contain the spread of SARS-CoV-2 [9]. In this context, study results not only highlight the importance of effective mouth-nose protection, but also how individual`s feeling of safety can be sustainably increased by a combination of different protective measures.

The use of disinfectants and transparent communication about the hygiene strategy increases an individual`s feeling of safety. Loss and colleagues [10] emphasize that current knowledge on many COVID-19 aspects is often uncertain or even lacking and stress the importance of risk communication. It can be assumed that hygiene and containment measures implemented during the first few weeks of the pandemic, thus being more visible to the public, might have influenced individuals` feeling of safety to a greater extent. Within early interventions at the beginning of the pandemic in 2020, taking of the body temperature was carried out in many areas of public life to determine whether the person concerned was suffering from fever, thus indicating whether one is infected with SARS-CoV-2. However, studies have found that fever occurs in only 45.4% of cases of mild and moderate COVID-19 [11, 12].

Taking into consideration the fact that around one-third of all SARS-CoV-2 infections are asymptomatic [13], from the present perspective, the taking of the body temperature alone cannot be considered as an indicator of SARS-CoV-2 infection, thus not be a stand-alone measurement to decide whether granting admission to an event or not.

This study shows, however, that especially these early pandemic measures seem to be fixed upon respondents’ memory and positively influence their feeling of safety, irrespective of scientific and medical usefulness of these measures. For example, body temperature measurement is rated as being more important than a negative SARS-CoV-2 test.

The same seems to apply for using disinfectants, being rated as most important, although scientific evidence emphasises the spread of SARS-CoV-2 via aerosols and droplets as the main way of transmission [14]. Due to the spread via aerosols and droplets and their transmission pathways depending on ventilation, occupation density and containment measures, indoor environments are particularly important for “catching the virus” [15]. From a medical perspective, wearing an effective mouth-nose protection or even a medical mask (FFP2 quality) is one of the most important components of a hygiene strategy, with single-layer masks being considered as unsuitable [9, 16, 17]. However, the most effective strategy is a multicomponent strategy (contact reduction, physical distance, wearing masks, adequate ventilation, staying home if sick, testing, vaccination, etc.)

To control the spread of the virus, especially the aerosol transmission, the abandonment of breaks and/or catering is another possible way. This containment strategy was rated ambivalently, i.e., being more important for women and older persons (66 years and older) than for men and younger persons (up to 40 years). It seems reasonable to assume that for young persons, an event is directly linked with food, drinks, and social conversations during breaks. Conversely, older persons seem to rather focus on the event itself and might therefore be fine without breaks and catering, and with the consequence of fewer contacts and less risk of infection.

## Limitations

The pandemic dynamics must be considered when evaluating the survey results. Our survey was conducted in November 2020. At that time, testing concepts for events, especially the use of rapid antigen tests, were hardly widespread. Furthermore, the study had been performed in a time period where vaccination and community testing with rapid tests had not been implemented in Germany. The prevalence of citizen testing, especially those offered for free, increased significantly during spring 2021. This changed not only the subjective perception of this measure compared to November 2020, but also opinions on hygiene and containment measures which also evolve in the light of political and medical requirements over time. Regularly recurring (longitudinal) surveys on the relevance and acceptance of hygiene and containment strategies are therefore necessary to obtain up-to-date findings for optimizing these concepts.

## Conclusion

Research in the field of cultural and sports events is particularly relevant to have an insight of how such events can take place with the greatest possible safety, but also with the highest possible acceptance of the measures by the visitors. It is essential to re-open the event sector as soon as possible, since not only the event itself is important, but also being together (i.e., creating a community feeling) is a special attraction of an event. As pointed out by observations from England, a ban of events does not make sense, on the contrary, illegal raves and unlicensed block parties have increased because of the ban [18]. It can therefore be assumed that young(er) persons act according to their desire for social events and far from any hygiene and/or containment measure, potentially resulting in an uncontrollable spread of SARS-CoV-2. This would not be the case for legally organized events with a proven hygiene and containment strategy. A step-by-step re-opening of the cultural event sector would therefore also contribute to an effective containment of the COVID-19 pandemic.

There is a need for further comprehensive public education to explain the spread of SARS-CoV-2, the symptoms associated with COVID-19 and corresponding hygiene and containment measures from a medical point of view. Comparing the subjective importance of hygiene and containment measures with objective evidence of effectiveness revealed a perception gap, i.e., measures such as taking the body temperature, obviously still leading to a high subjective feeling of safety, but in fact not being a good measure from a medical point of view. Thus, body temperature measurement, for example, should only be applied in combination with other, more effective measures. By contrast, self-reported measures such as unsupervised self-tests and health questionnaires might be less relevant and reliable, thus not being the only criterion for admission to an event.

For cultural event providers, paying attention to comprehensible and transparent communication of hygiene measures is necessary, ideally providing this information already before and during the purchase of a ticket, thereby appropriately addressing their target group.

For example, if the use of disinfectants is more important to women than men, this should be considered in defining and implementing target-group-specific events. The same applies for considering an abandonment of breaks and catering, which might be rather unimportant to older adults (aged 66 and older), but should not be considered for events aimed mainly at younger adults (aged 35 or younger). On the contrary, event concepts for younger adults should rather define an adequate concept for one-way systems and queuing to value the importance of it for this target group.

However, further research is needed to address the readiness of visitors to finance measures and a more in-depth evaluation regarding socio-demographic differences. This should be done in regular cycles with the same sample to identify possible changes in the attitudes of the respondents due to the dynamic pandemic situation.

## Data Availability

Data may be made available by contacting the corresponding author.

## Abbreviations

M: Mean
RKI: Robert-Koch Institute
WHO: World Health Organization
SE: Standard Error
i.e.: Id est (so to say)
e.g.: Exempli gratia (for example)
n: Number of participants (sample size)
F: *F*-Value
p: *p*-Value
